# Stroke among highly active antiretroviral therapy-naive people living with the human immunodeficiency virus in China: a retrospective study of the characteristics, risk factors, and prognosis

**DOI:** 10.1186/s12879-021-06989-6

**Published:** 2022-01-04

**Authors:** Ling Zhang, Yu Wang, Qiuhua Xu, Wei Zhang, Hongyuan Liang, Liang Wu, Liang Ni, Guiju Gao, Di Yang, Hongxin Zhao, Jiang Xiao

**Affiliations:** Clinical and Research Center of Infectious Diseases, Beijing Ditan Hospital, Jingshun East Street, Chaoyang District, Beijing, 100015 China

**Keywords:** PLWH, HAART-naive, Stroke, Risk factors

## Abstract

**Background:**

We aimed to clarify the characteristics, risk factors, and prognosis of stroke among HAART-naive people living with HIV (PLWH) in China.

**Methods:**

We selected HAART-naive PLWH admitted to Beijing Ditan Hospital, Capital Medical University, from 1 January 2009 to 31 December 2019. Demographic and clinical data were obtained by searching an anonymous electronic case system. Descriptive analysis and logistic regression and Cox proportional hazard models were used to determine the characteristics and predictors of stroke among all HAART-naive PLWH and evaluate the risk factors of mortality in HAART-naive PLWH with stroke.

**Results:**

Stroke was diagnosed in 105 cases (3.7%) of 2867 HAART-naive PLWH. Multivariate logistic regression indicated that age of 30–55 years (OR 1.903, 95% CI 1.005–3.603, p = 0.048), age of ≥ 55 years (OR 4.104, 95% CI 1.928–8.737, p < 0.001), and CD4 count of < 200 cells/µL (OR 2.005, 95% CI 1.008–3.985, p = 0.047) were associated with increased odds of stroke. Diabetes (OR 3.268, 95% CI 1.744–6.125, p < 0.001), hypertension (OR 2.301, 95% CI 1.425–3.717, p = 0.001), syphilis (OR 2.003, 95% CI 1.300–3.089, p = 0.002), and complicated AIDS-defining CNS diseases (OR 7.719, 95% CI 4.348–13.703, p < 0.001) were risk factors for stroke. Of the 105 stroke patients, 12 (11.4%) died during hospitalisation, and the risk factors for mortality among patients with stroke were age of > 65 years (AHR: 8.783, 95% CI 1.522–50.668, p = 0.015), complicated severe pneumonia (AHR: 3.940, 95% CI 1.106–14.029, p = 0.034), and AIDS-defining CNS diseases (AHR: 19.766, 95% CI 3.586–108.961, p = 0.001).

**Conclusions:**

For HAART-naive people living with HIV (PLWH), stroke occurred in various age groups, and early screening for stroke, timely intervention for risk factors among patients in various age groups, and controlling the CD4 count are extremely important in reducing the burden of stroke.

## Introduction

The development of highly active antiretroviral therapy (HAART) has transformed fatal HIV infection into a chronic and controllable disease, and the life expectancy of people living with HIV (PLWH) receiving antiretroviral regimens (ARV) has greatly increased [1]. However, several PLWH did not initiate HAART in China due to unawareness of HIV infection, social discrimination, psychological pressure, and poor compliance [2], until they were first hospitalised for opportunistic infections. For some cases, HIV infection was confirmed based on routine screening on admission due to comorbidities, which indicated that different comorbidities, including AIDS-defining and non-AIDS-defining events, should be considered in HAART-naive PLWH. Stroke is one of the common non-AIDS-defining events [3], which places a huge economic burden on families of patients and the society at large.

Previous studies have indicated that stroke is likely to occur due to residual chronic inflammation [4]. However, the predictors of stroke and the subsequent risk factors of mortality among HAART-naive PLWH have not been elucidated. This study aimed to report the characteristics and predictors of stroke among all HAART-naive PLWH and evaluate the risk factors of mortality in HAART-naive PLWH with stroke, which will help clinicians perform early screening and timely interventions to reduce the occurrence of stroke and improve prognosis.

## Methods

### Ethics

Consistent with the principles of the Helsinki Declaration, this retrospective study was approved by the Human Science and Ethics Committee of Beijing Ditan Hospital, Capital Medical University. The clinical data were obtained anonymously from the electronic medical records system and analysed, and the Human Scientific Ethics Committee waived the requirement for informed consent.

### Study participants

We selected HAART-naive PLWH admitted to Beijing Ditan Hospital, Capital Medical University, between January 1, 2009, and December 31, 2019. Only patients with complete demographic and clinical data, including sex, age, history of smoking and drinking, and route of HIV infection, were included in our study. Medical history, including previous diagnoses of hypertension, diabetes, and syphilis and the medical diagnosis on discharge from the hospital were required. Participants were excluded if they were pregnant women, were aged ≤ 18 years, foreigners, had experience with HAART, and had missing information.

### Definitions and diagnosis

The laboratory diagnosis of HIV infection was mainly based on serological screening tests and confirmed by confirmatory tests before the diagnosis of HIV infection [5]. Serological screening tests were performed by ELISA, and a confirmatory test was performed based on western blotting (WB) when the result was positive.

Stroke was defined as an acute episode of focal dysfunction of the brain, retina, or spinal cord with typical symptoms or a local infarction or haemorrhage detected on imaging (CT or MRI) associated with symptoms [6]. The pathological subtypes of stroke include ischaemic stroke and haemorrhagic stroke; lacunar infarction is also included in ischaemic stroke [6]. However, the clinical manifestations and prognosis of lacunar infarction were relatively mild, and this study separately listed lacunar infarction from ischaemic stroke.

The diagnostic criteria of diabetes were as follows: glycosylated haemoglobin of ≥ 6.5%, random plasma glucose of ≥ 11.1 mmol/l, fasting blood glucose of ≥ 7.0 mmol/l, or venous plasma glucose of ≥ 11.1 mmol/l at 2 h after OGTT [7].

In the absence of antihypertensive drugs, systolic blood pressure of ≥ 140 mmHg and diastolic blood pressure of ≥ 90 mmHg. Previous history of hypertension or antihypertensive drugs were used, even if the blood pressure does not reach the above level, hypertension should be diagnosed based on history [8].

Syphilis is a chronic systemic sexually transmitted disease caused by Treponema pallidum, which is a curable sexually transmitted disease. A presumptive serologic diagnosis of syphilis was possible based on non-treponemal tests (i.e. Venereal Disease Research Laboratory [VDRL] and rapid plasma reagin [RPR]) and treponemal tests (i.e., fluorescent treponemal antibody absorbed [FTA-ABS] and T. pallidum particle agglutination [TPPA], et al.) [9].

The diagnosis of opportunistic infections was based on the guidelines proposed by the National Institutes of Health (NIH) [9].

Severe pneumonia was defined as one or more co-infections of bacteria, fungi, and viruses associated with serious pulmonary infection and immunosuppression, which met one of the major conditions or two secondary conditions. The major conditions were as follows: (1) mechanical ventilation was needed and (2) vasoactive agents were still needed after active fluid resuscitation for septic shock. The secondary conditions were as follows: (1) respiratory rate of > 30 times/min, (2) PaO_2_/FiO_2_ of < 250 mmHg, and (3) systolic blood pressure of < 90 mmHg and diastolic blood pressure of < 60 mmHg, which required active fluid resuscitation [10].

AIDS-defining central nervous system (CNS) diseases refer to opportunistic infections or non-Hodgkin lymphoma in the central nervous system, including cryptococcal and tuberculous meningitis, Toxoplasma gondii encephalitis, progressive multifocal leukoencephalopathy (PML), and non-Hodgkin lymphoma [11].

### Data collection

Demographic and clinical data, including sex, age, history of smoking and drinking, and route of HIV infection, were obtained by searching the anonymous electronic case system. The criterion for cigarette smoking history was smoking for more than 5 years [12], and that for alcoholic drinking history was alcohol intake of > 40 g/day by men or > 20 g/day by women for more than 5 years [13]. The baseline laboratory tests included the measurement of CD4 count, haemoglobin level, and blood lipid panel, including total cholesterol (TC), triglyceride (TG), low-density lipoprotein (LDL), and high-density lipoprotein (HDL).

MRI or CT was performed only for patients with typical symptoms of stroke, such as sudden unilateral weakness, numbness, visual loss, diplopia, altered speech, ataxia, and non-orthostatic vertigo [6]. Patients with symptoms of CNS infections also underwent neuroimaging.

We also recorded medical history including hypertension, diabetes, syphilis, and medical diagnosis when discharged from the hospital.

Lumbar puncture was performed to identify CNS diseases based on one of the following conditions: (1) symptoms and signs of CNS infections; (2) a space-occupying lesion based on CT or MRI screening, (3) some special pathogen infections, including haematogenous disseminated pulmonary tuberculosis and haematic cryptococcal antigen positivity. CNS opportunistic infections [14], including tuberculous meningitis, cytomegalovirus encephalitis, progressive multifocal leukoencephalopathy (PML), meningitis cryptococcal, and toxoplasmosis encephalopathy, were evaluated.

The prognosis of these patients at discharge was recorded as death or survival.

### Data analysis

Descriptive analysis using percentages was conducted in this study. Histograms were used to illustrate the proportion of patients with stroke and stroke mortality in HAART-naive PLWH stratified by age and CD4 categories.

SPSS 24.0 (SPSS Institute, Chicago IL, USA) was used for the statistical analysis. Categorical variables were expressed as percentages and compared using the chi-squared test, while continuous variables were presented as median, maximum, and minimum values.

The correlations between traditional risk factors and stroke were evaluated using univariate logistic regression models, and multivariate logistic regression models were used to analyse the predictors which were statistically significant (p < 0.1).

The Cox proportional hazard model was applied to evaluate the hazards of in-hospital mortality in HAART-naive PLWH with stroke, and Kaplan–Meier survival curves were used to assess the survival of HAART-naive PLWH with or without severe pneumonia.

Bilateral alpha was set to 0.05, and statistical significance was set at p < 0.05.

## Results

### Demographic and clinical characteristics

The study enrolled 2867 HAART-naive PLWH admitted to Beijing Ditan Hospital affiliated to Capital Medical University between January 1, 2009, and December 31, 2019. The demographic and clinical characteristics of the patients are listed in Table 1.

**Table 1:** Baseline demographics, clinical and laboratory features of stroke among HAART-naive people living with HIV (PLWH)

Variables	Total	Stroke-free	Stroke	*p*	Types of Stroke				*p*
					Ischemic infarction	Hemorrhagic infarction	Lacunar infarction	Unknown/Other	
Patients number (%)	2867 (100)	2762 (100)	105 (100)	–	59 (100)	5 (100)	34 (100)	7 (100)	–
Demographic data									
Age (years)^a^	37 (18–83)	38 (18–79)	47 (20–83)	–	46 (22–70)	39 (24–51)	51 (20–83)	44 (32–54)	–
Age < 30 years	724 (25.3)	712 (25.8)	12 (11.4)	< 0.001	9 (15.3)	1 (20.0)	2 (5.9)	0	0.023^b^
30 ≤ Age < 55 years	1846 (64.4)	1781 (64.5)	65 (61.9)		36 (61.0)	4 (80.0)	18 (52.9)	7 (100.0)	
Age ≥ 55 years	297 (10.4)	269 (9.7)	28 (26.7)		14 (23.7)	0	14 (41.2)	0	
Gender-male (%)	2587 (90.2)	2493 (90.3)	94 (89.5)	0.803	52 (88.1)	3 (60.0)	32 (94.1)	7 (100.0)	0.138^c^
Cigarette smoking history (%)	892 (31.1)	848 (30.7)	44 (41.9)	0.015	24 (40.7)	2 (40.0)	14 (41.2)	4 (57.1)	0.871^c^
Alcoholic drinking history (%)	770 (26.9)	739 (26.8)	31 (29.5)	0.530	18 (30.5)	2 (40.0)	9 (26.5)	2 (28.6)	0.933^c^
Transmission route									
Homosexual (%)	1728 (60.6)	1671 (60.5)	67 (63.8)	0.611^c^	38 (64.4)	2 (40.0)	22 (64.7)	5 (71.4)	0.847^b^
Heterosexual (%)	811 (28.3)	787 (28.5)	24 (22.9)		13 (22.0)	3 (60.0)	7 (20.6)	1 (14.3)	
Blood transfusion (%)	271 (9.5)	259 (9.4)	12 (11.4)		7 (11.9)	0	4 (11.8)	1 (14.3)	
Intravenous drug (%)	47 (1.6)	45 (1.6)	2 (1.9)		1 (1.7)	0	1 (2.9)	0	
Laboratory results									
CD4 < 200 cells/µL (%)	2378 (82.9)	2283 (82.7)	95 (90.5)	0.037	51 (86.4)	5 (100.0)	32 (94.1)	7 (100.0)	0.262
TC > 6.2 mmol/L (%)	34 (1.2)	33 (1.2)	1 (1.0)	1.000^c^	0	0	1 (2.9)	0	0.517^b^
TG > 2.3 mmol/L (%)	318 (11.1)	304 (11.0)	14 (13.3)	0.456	7 (11.9)	1 (20.0)	4 (11.8)	2 (28.6)	0.688^c^
HDL < 0.96 mmol/L (%)	2451 (85.5)	2357 (85.3)	94 (89.5)	0.232	53 (89.8)	4 (80.0)	30 (88.2)	7 (100.0)	0.571^c^
LDL > 4.2 mmol/L (%)	28 (1.0)	28 (1.0)	0	0.595^b^	–	–	–	–	
Epidemiological data									
Complications									
Diabetes (%)	110 (3.8)	94 (3.4)	16 (15.2)	< 0.001	9 (15.3)	1 (20.0)	5 (14.7)	1 (15.2)	0.993^c^
Hpertension (%)	351 (12.2)	318 (11.5)	33 (31.4)	< 0.001	16 (27.1)	3 (60.0)	12 (35.3)	2 (28.6)	0.475^c^
Syphilis (%)	589 (20.5)	553 (20.0)	36 (34.3)	< 0.001	25 (42.4)	0	9 (26.5)	2 (28.6))	0.074^b^
Severe pneumonia (%)	261 (9.1)	246 (8.9)	15 (14.3)	0.060	4 (6.8)	0	8 (23.5)	3 (42.9)	0.017^b^
AIDS-defining CNS diseases (%)	105 (3.7)	86 (3.1)	19 (18.1)	< 0.001	10 (16.9)	2 (40.0)	5 (14.7)	2 (28.6)	0.553^c^
Opportunistic infections									
CMV (%)	800 (27.9)	767 (27.8)	33 (31.4)	0.412	19 (32.2)	0	15 (44.1)	4 (57.1)	0.070^b^
PCP (%)	1034 (36.1)	994 (36.0)	40 (38.1)	0.659	22 (37.3)	1 (20.0)	13 (38.2)	4 (57.1)	0.611^c^
TB (%)	731 (25.5)	712 (25.7)	19 (18.1)	0.180	8 (13.6)	2 (40.0)	8 (23.5)	1 (14.3)	0.409^c^
Cryptococcosis (%)	185 (6.5)	177 (6.4)	8 (7.6)	0.620	1 (1.7)	0	5 (14.7)	2 (28.6)	0.022^b^
Death (%)	188 (6.6)	176 (6.3)	12 (11.4)	0.040	5 (8.5)	2 (40.0)	4 (11.8)	1 (14.3)	0.350^c^

*HDL* high-density lipoprotein; *LDL* low-density lipoprotein; *TC* total cholesterol; *TG* triglyceride; *CMV* cytomegalovirus; *TB* tuberculosis; *PCP* pneumocystis pneumonia; *CNS* central nervous system

^a^Evaluation based on minimum and maximum values

^b^Likelihood test

^c^Continuous correction method

The average age of the 2867 patients was 37 years (range 18–83 years). The number of patients aged < 30, 30–55, and ≥ 55 years were 724 (25.3%), 1846 (64.4%), and 297 (10.4%), respectively. The number of patients with CD4 cell counts of < 200 cells/µL was 2387 (82.9%).

Of 2867 patients, 3.7% (105 cases) were diagnosed with stroke; the proportions of the ischaemic, haemorrhagic, lacunar, and unknown subtypes were 56.2% (59 cases), 4.8% (5 cases), 32.3% (34 cases), and 6.7% (7 cases), respectively. (Table 1).

### The age and CD4 stratifications for stroke

**Fig. 1:**
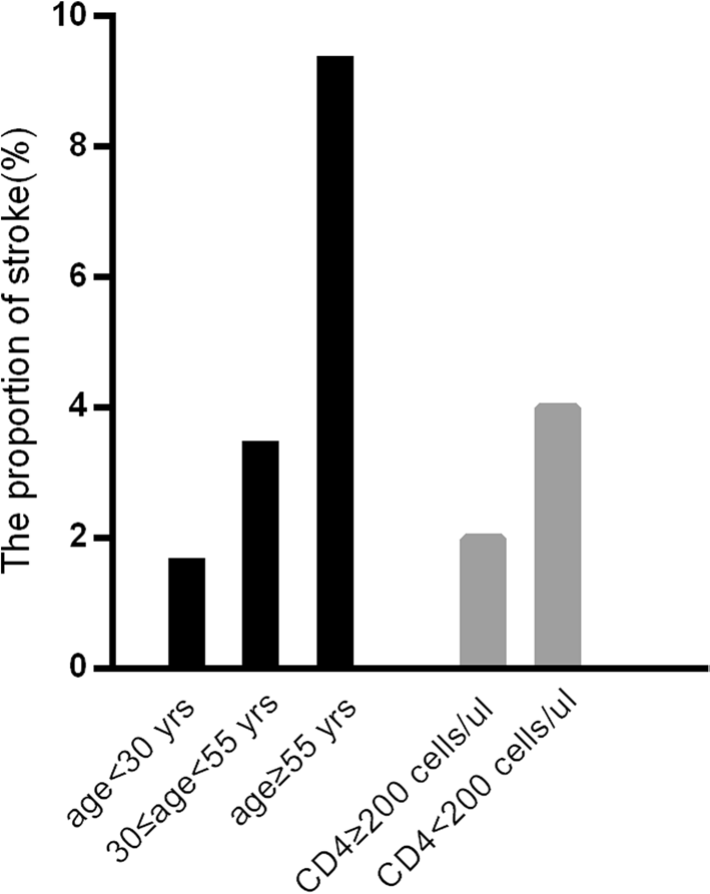
Histograms illustrating the age and CD4 stratifications. The proportions of stroke patients aged < 30, 30–55, and ≥ 55 years were 1.7%, 3.5%, and 9.4%, respectively. Among patients with CD4 cell counts of ≥ 200 and < 200 cells/µL, the proportions of stroke were 2.0% and 4.0%, respectively

The proportions of stroke patients aged < 30, 30–55, and ≥ 55 years were 1.7%, 3.5%, and 9.4%, respectively. Among patients with CD4 cell counts of ≥ 200 and < 200 cells/µL, the proportions of those with stroke were 2.0% and 4.0%, respectively (Fig. 1).

### Predictors of stroke among HAART-naive PLWH

As shown in Table 2, the multivariate logistic regression analysis model revealed that age of 30–55 years (odds ratio [OR]: 1.903, 95% CI 1.005–3.603, p = 0.048), age of ≥ 55 years (OR 4.104, 95% CI 1.928–8.737, p < 0.001), and CD4 count of < 200 cells/µL (OR 2.005, 95% CI 1.008–3.985, p = 0.047) were associated with increased odds of stroke. Diabetes (OR 3.268, 95% CI 1.744–6.125, p < 0.001), hypertension (OR 2.301, 95% CI 1.425–3.717, p = 0.001), syphilis (OR 2.003, 95% CI 1.300–3.089, p = 0.002), and AIDS-defining CNS diseases (OR 7.719, 95% CI 4.348–13.703, p < 0.001) were risk factors for stroke.

**Table 2:** Risk factors for stroke by logistic regression analysis models among HAART-naive people living with HIV (PLWH)

Variables	Unadjusted		Adjusted	
	OR (95% CI)	*p*	OR (95% CI)	*p*
Age < 30 years	1		1	
30 ≤ Age < 55 years	2.165 (1.163–4.033)	0.015	1.903 (1.005–3.603)	0.048
Age ≥ 55 years	6.176 (3.096–12.321)	< 0.001	4.104 (1.928–8.737)	< 0.001
Gender-Male	0.922 (0.488–1.744)	0.803	–	–
Cigarette smoking history	1.628 (1.096–2.419)	0.016	1.339 (0.882–2.032)	0.170
Alcoholic drinking history	1.147 (0.748–1.759)	0.530	–	–
Laboratory results				
CD4 < 200 cells/µL	1.993 (1.031–3.853)	0.040	2.005 (1.008–3.985)	0.047
TC > 6.2 mmol/L	0.795 (0.108–5.870)	0.822	–	–
TG > 2.3 mmol/L	1.244 (0.700–2.211)	0.457	–	–
HDL < 0.96 mmol/L	1.468 (0.779–2.766)	0.235	–	–
Complications				
Diabetes	5.103 (2.884–9.028)	< 0.001	3.268 (1.744–6.125)	< 0.001
Hpertension	3.523 (2.295–5.406)	< 0.001	2.301 (1.425–3.717)	0.001
Syphilis	2.084 (1.378–3.152)	0.001	2.003 (1.300–3.089)	0.002
Severe pneumonia	1.705 (0.972–2.990)	0.063	1.354 (0.744–2.462)	0.321
AIDS-defining CNS diseases	6.875 (4.001–11.812)	< 0.001	7.719 (4.348–13.703)	< 0.001
Opportunistic infections				
CMV	1.192 (0.783–1.815)	0.413	–	–
PCP	1.095 (0.733–1.635)	0.659	–	–
TB	0.637 (0.385–1.054)	0.079	0.636 (0.376–1.077)	0.092
Cryptococcosis	1.204 (0.576–2.517)	0.621	–	–

*HDL* high-density lipoprotein; *LDL* low-density lipoprotein; *TC* total cholesterol; *TG* triglyceride; *CMV* cytomegalovirus; *TB* tuberculosis; *PCP* pneumocystis pneumonia; *CNS* central nervous system; *OR* odds ratio

### Mortality of stroke in HAART-naive PLWH stratified by age and CD4 category

**Fig. 2:**
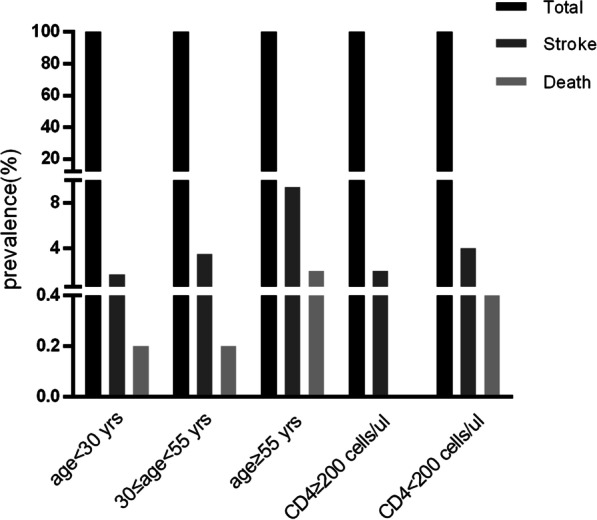
Histograms showing the mortality rates of stroke in HAART-naive people living with HIV (PLWH) stratified by age and CD4 count. Mortality rates associated with ages of < 30, 30–55, and ≥ 55 years were 0.2% (2 cases), 0.2% (4 cases), and 2.0% (6 cases), respectively. The mortality rates associated with CD4 counts of < 200 and ≥ 200 cells/µL were 0.5% (12 cases) and 0, respectively

Among 2867 HAART-naive PLWH, 105 were diagnoses with stroke, of which 11.4% (n = 12) died. The mortality rates stratified by age and CD4 count are shown in Fig. 2.

### Survival analysis

Survival analysis of 105 HAART-naive PLWH with stroke was further conducted to identify the risk factors of mortality in HAART-naive PLWH with stroke. The multivariate Cox regression model indicated that age of > 65 years (adjusted hazard ratio [AHR]: 8.783, 95% CI 1.522–50.668, p = 0.015), severe pneumonia (AHR: 3.940, 95% CI 1.106–14.029, p = 0.034), and AIDS-defining CNS diseases (AHR: 19.766, 95% CI 3.586–108.961, p = 0.001) were associated with an increased risk of death among HAART-naive PLWH with stroke (Table 3).

**Table 3:** Risk factors for mortality by Cox proportional hazard model among HAART-naive people living with HIV (PLWH) with stroke

Variables	Unadjusted		Adjusted	
	HR (95%CI)	*p*	HR (95%CI)	*p*
Age > 65 years	4.476 (1.335–15.008)	0.015	8.783 (1.522–50.668)	0.015
Gender-Male	1.104 (0.142–8.592)	0.925	–	–
Cigarette smoking history	0.805 (0.241–2.681)	0.723	–	–
Alcoholic drinking history	0.264 (0.034–2.051)	0.203	–	–
Laboratory results				
CD4 < 50 cells/µL	0.599 (0.187–1.922)	0.389	–	–
TC > 6.2 mmol/L	0.049 (0.000–141.646)	0.869	–	–
TG > 2.3 mmol/L	1.197 (0.260–5.523)	0.818	–	–
HDL < 0.96 mmol/L	1.183 (0.151–9.243)	0.873	–	–
Complications				
Diabetes	2.117 (0.571–7.844)	0.262	–	–
Hpertension	3.236 (1.002–10.248)	0.046	2.763 (0.731–10.448)	0.134
Syphilis	0.398 (0.087–1.822)	0.235	–	–
Severe pneumonia	5.087 (1.628–15.890)	0.005	3.940 (1.106–14.029)	0.034
AIDS-defining CNS diseases	5.127 (1.648–15.952)	0.005	19.766 (3.586–108.961)	0.001
Opportunistic infections				
CMV	1.614 (0.498–5.236)	0.425	–	–
PCP	1.361 (0.434–4.271)	0.597	–	–
TB	0.730 (90.156–3.410)	0.689	–	–
Cryptococcosis	3.173 (0.686–14.686)	0.140	–	–

*HDL* high-density lipoprotein; *LDL* low-density lipoprotein; *TC* total cholesterol; *TG* triglyceride; *CMV* cytomegalovirus; *TB* tuberculosis; *PCP* pneumocystis pneumonia; *CNS* central nervous system; *HR* hazard ratio

**Fig. 3:**
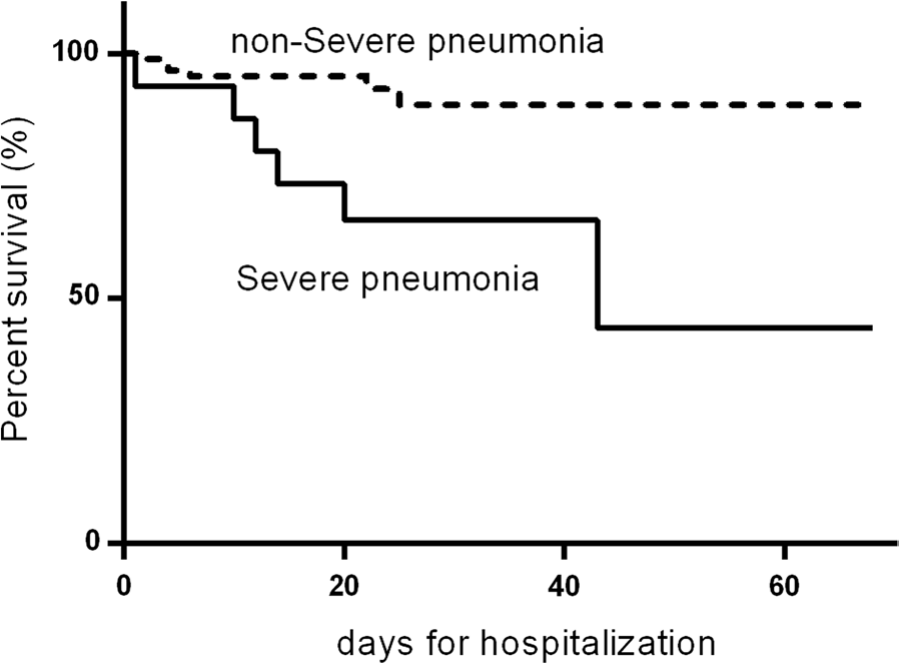
Kaplan–Meier survival curves for HAART-naive people living with HIV (PLWH) with/without severe pneumonia

To further elucidate the effect of the above results, we plotted Kaplan–Meier survival curves based on the presence or absence of severe pneumonia, and log-rank testing demonstrated the diversity of the two groups, indicating that severe pneumonia was associated with increased mortality in HAART-naive PLWH with stroke (Fig. 3).

## Discussion

Stroke is a common geriatric disease, and it is a common cause of disability and death in the general population [15]. According to a previous study, stroke was also a common non-AIDS-defining event (NADE) among PLWH receiving HAART [3], which was caused by residual chronic inflammation. In HAART-naive PLWH, the characteristics, risk factors, and prognosis of stroke have rarely been reported [16]. Our study is the first to elucidate the characteristics and risk factors of stroke, as well as predictors of mortality, in HAART-naive PLWH, and the findings will help clinicians to provide timely interventions for these patients.

Our previous study indicated that [2], HIV/AIDS patients admitted to Beijing Ditan Hospital, which is the largest HIV/AIDS referral centre in China, were representative of all the provinces in China, and the clinical characteristics and conclusions in this paper can be generalised to some extent in China.

Our study found that ischaemic infarction accounted for the highest proportion of HAART-naive PLWH with stroke (Table 1). In these patients, ischaemic stroke may be caused by cytokines released from HIV-infected cells, which make blood vessels prone to vasospasm, atherosclerosis, and thrombosis by producing endothelial vasodilators and changing vascular activity [17].

Matthias et al*.* found that [18]increasing age was one of the irreversible risk factors for the development of stroke, which may be related to the increased expression of inflammatory factors associated with increasing age. Virani et al*.* indicated that [15], stroke is likely to occur in older patients in the general population. In this study, we found that age was an independent risk factor for stroke. Wang et al*.* demonstrated that the average age of stroke occurrence in China was 66.4 years in the general population [19]; among HAART-naive PLWH, the average age of stroke was 47 years, which indicated that stroke can occur in younger HAART-naive PLWH and may be related to HIV-mediated chronic immune activation resulting in the accelerated aging process of AIDS patients [20]. Physicians should be aware that stroke can occur in younger HAART-naive PLWH of different ages and not only in older adults.

In this study, we found that CD4 count of < 200 cells/µL was an independent predictor of stroke, which was consistent with the results reported by Venkat et al*.* [21]. In HAART-naive PLWH, a lower CD4 count indicates severe immunosuppression, and the HIV may promote the formation of atherosclerosis by activating endothelial cells, as well as immune cells, and increasing the number of atherogenic immune cells in vivo [22]. For patients with CD4 counts of < 200 cells/µL, HAART should be initiated as soon as possible to inhibit viral replication and improve immune status to reduce the risk of occurrence of stroke. Elevated cytokine levels are considered a major problem in HIV pathogenesis [23], and HIV-mediated chronic inflammation increases the risk of stroke.

As a traditional risk factor for stroke, diabetes has been reported in the general population, and elevated plasma glucose in vivo can lead to microvascular and macrovascular complications, including stroke and cardiovascular and peripheral vascular diseases [24]. Pandian et al*.* [25] reported that glycaemia control is one of the methods of evidence-based secondary prophylaxis for stroke. In this study, we also found that diabetes was another independent risk factor for the occurrence of stroke among HAART-naive PLWH, which suggests that timely clinical interventions for diabetes in HAART-naive PLWH may be beneficial for stroke prevention in this population.

Hypertension was previously reported as an important risk factor for stroke, and more than half of stroke cases worldwide were attributed to hypertension [26]. This study arrived at a similar conclusion that hypertension may increase the risk of stroke in HAART-naive PLWH, indicating that active treatment of hypertension may help reduce the risk of developing cerebrovascular disease.

The invasion of the central nervous system by *Treponema pallidum* can occur at any stage of syphilis infection, which causes extensive meningeal and perivascular space dilatation, lymphocyte infiltration, and intimal hyperplasia, resulting in stenosis, vascular occlusion, and ischaemic stroke [27]. In this study, we also found that syphilis was associated with stroke, which indicated that early anti-syphilis treatment was necessary for HAART-naive PLWH with syphilis infection.

It has been reported that stroke is a common complication in patients with chronic meningitis, such as tuberculous meningitis and cryptococcal meningitis, and one of the mechanisms is that cerebral vessels cross the exudate at the base of the brain, resulting in vascular strangulation and the development of vasculitis with inflammation, spasm, contraction, and, ultimately, thrombosis [28]. In this study, we concluded that AIDS-defining CNS disease was an independent predictor of an increased risk of stroke. This indicates that early diagnosis of such diseases and appropriate therapy are key to reducing the risk of stroke.

Studies have indicated that dyslipidaemia [29] and smoking [30] are modifiable risk factors for cerebrovascular disease. Our findings are inconsistent with those of these studies. However, Okada et al*.* reported that smoking habits were associated with the development of stroke, particularly thrombosis, but this relationship was not obvious and may be modified by other related factors [31]. Takeya et al*.* indicated that reports relating cigarette smoking to stroke were also mixed [32], which was similar to our conclusion.

Wsttanakit et al*.* demonstrated in a large epidemiological study that dyslipidaemia was weakly and inconsistently associated with stroke [33], which was consistent with our conclusion. This suggests that these variables were considered confounders when assessing risk factors of stroke, while other predictors such as CD4 count and age were the main risk factors in multivariate logistic regression models.

Respiratory infections have been reported to be risk factors for mortality among the general population with stroke [34], and we found that severe pneumonia was an independent predictor of mortality among HAART-naive PLWH with stroke. Physicians should be aware that active treatment of severe pneumonia can help reduce mortality in these patients. AIDS-defining CNS diseases are also risk factors for death in patients with HAART-naive HIV/AIDS complicated with stroke, which suggests that early screening of nervous system diseases for HAART-naive PLWH is necessary to improve prognosis.

A European multicentre study of stroke found that age was an independent risk factor for increased mortality [35]. In this study, we reached a similar conclusion that age of > 65 years was an independent risk factor for mortality among HAART-naive PLWH with stroke, which showed that early diagnosis and active treatment of stroke were necessary to improve prognosis among older AIDS patients.

Our study had some limitations. First, potential bias in the retrospective study was inevitable. Second, in this study, we found that stroke occurred in 3.7% of HAART-naive PLWH, which represented the prevalence of admitted HAART-naive PLWH, but not among all HAART-naive PLWH in China. HAART-naive PLWH with and without mild CNS symptoms were not admitted to the hospital, and patients with severe CNS symptoms may be dead on the way to the hospital, and the prevalence of stroke in this study was higher based on the aforementioned reasons. Moreover, patients with incomplete data were not included in our study, which may have also resulted in a high prevalence of stroke.

In conclusion, we found that stroke could occur among HAART-naive PLWH, distributing in different CD4 levels and age groups. We also found that hypertension, diabetes, syphilis, and AIDS-defining CNS diseases were predictors of stroke, while age of > 65 years, AIDS-defining CNS diseases, and severe pneumonia were risk factors for death among these patients. Our results indicate that early screening and early interventions targeted at these predictors are important for reducing the burden of stroke among HAART-naive PLWH.

## Conclusion

Our study is the first to elucidate the characteristics and risk factors of stroke in HAART-naive PLWH, as well as the predictors of mortality in these patients with stroke, which will help clinicians provide timely interventions for these patients.

## Data Availability

The datasets used and/or analysed during the current study are available from the corresponding author upon reasonable request.

## Abbreviations

AIDS: Acquired immunodeficiency syndrome
ARV: Antiretroviral regimens
CMV: Cytomegalovirus
CNS: Central nervous system
HAART: Highly Active Antiretroviral Therapy
HDL: High-density lipoprotein
HR: Hazard ratio
LDL: Low-density lipoprotein
NADE: Non-AIDS-defining-event
OR: Odds ratio
PCP: Pneumocystis Pneumonia
PLWH: People living with HIV
PML: Progressive multifocal leukoencephalopathy
TB: Tuberculosis
TC: Total cholesterol
TG: Triglyceride
